# Hepatitis B Virus Infections and Associated Factors among Pregnant Women Attending Antenatal Care Clinic at Deder Hospital, Eastern Ethiopia

**DOI:** 10.1371/journal.pone.0166936

**Published:** 2016-11-29

**Authors:** Abdi Umare, Berhanu Seyoum, Tesfaye Gobena, Tamirat Haile Mariyam

**Affiliations:** 1 Department of Medical Laboratory, Deder Hospital, Deder, Ethiopia; 2 Department of Microbiology Sciences, College of Health and Medical Sciences, Haramaya University, Harar, Ethiopia; 3 Department of Public Health, College of Health and Medical Sciences, Haramaya University, Harar, Ethiopia; 4 Ethiopian Red Cross Society Blood Bank, Harar Branch, Ethiopia; Centre de Recherche en Cancerologie de Lyon, FRANCE

## Abstract

**Background:**

Hepatitis B virus (HBV) infection is a serious public health problem worldwide. Reports have shown that 68,600 people die of HBV infection and more than 300,000 deaths due to liver cancer secondary to hepatitis B every year globally. Women who are infected with HBV can vertically transmit the infection to their infants. This study aims to determine the prevalence of HBV infection and associated factors among pregnant women.

**Methods and Findings:**

A hospital-based cross-sectional study was conducted among pregnant women who attended antenatal care clinic (ANC) for routine pregnancy check-up between 18 March 2015 and 15 May 2015. Data were collected by face to face interview using a pre-tested questionnaire. Serum was withdrawn for each study subject and used to test for Hepatitis B Surface Antigen (HBsAg) by an enzyme linked immunosorbent assay (ELISA) test kit. Binary logistic regression analysis was used to examine the association between explanatory variables and outcome variable. The prevalence of HBV infection was found to be 6.9%. Interestingly, the history of abortion (AOR 10.9; 95% CI: 2.2–53.9), nose piercing (AOR 9.1; 95% CI: 1.34–61.79), surgical procedure (AOR 12.8; 95% CI: 1.68–97.06) and history of multiple sexual partners (AOR 16.8; 95% CI: 3.18–89.06) were significant predictors of HBV infection.

**Conclusions:**

This study determined that the prevalence of HBV infection among pregnant women was 6.9%, implying that it is high-intermediate endemic area, which is important public health issue needs to be addressed. History of abortion, nose piercing, surgical procedures and multiple sexual partners were significantly associated with this viral infection. Accordingly we advocate that health education programs on the mode of HBV transmission, high-risk behaviors and methods of preventions should be instituted at antenatal care clinics to raise the awareness of mothers and limit the spread of infection. It is also advisable to implement nosocomial infection prevention strategies to prevent the transmissions of HBV through health care related activities such as surgical procedures. Furthermore, all pregnant women should be screened for HBV, treated if necessary to reduce their viral loads and their children vaccinated at birth with the single-dose hepatitis B vaccine to break the cycle of mother-to-child transmission.

## Background

Hepatitis B infection is caused by the hepatitis B virus (HBV). It is an enveloped DNA virus that infects the liver and causes hepatocellular necrosis and inflammation [1]. HBV infection is one of a serious public health problem worldwide and it is 50–100 times more contagious than HIV [2, 3]. Many of the carriers do not realize that they are infected with the virus rendering the HBV to be known as a “silent killer” [4].

Worldwide, it is estimated that 240 million people are chronically infected with hepatitis B [5]. The largest number of people living with chronic HBV live in the Western Pacific region (over 95 million) followed by the African region (over 75 million) [6]. Recent reports demonstrated that 68,600 people die of HBV infection and more than 300,000 deaths due to liver cancer secondary to hepatitis B every year globally [7]. Perinatal and early childhood transmissions are the main routs of HBV infection in high and intermediate endemic areas [8]. Administering HBV vaccination to 90% of newborns within 24 hours from birth would prevent 84% of global HBV-related deaths [9].

In Ethiopia, Expanded Programme on Immunization (EPI) was launched in 1980 with the objective of achieving universal child immunization by 1990. However, the immunization policy was updated in 2007 which enrolled childhood immunization against HBV in a penta-valent form at the age of 6, 10 and 14 weeks after birth [10]. It has been noticed that the risk of HBV transmission decreases in a setting where there is a periodic perinatal HBV screening, immunoprophylaxis given to infants born to HBV infected mothers and hepatitis B vaccine administered both to high risk mothers and to all newborn infants [11]. Antenatal screening for HBsAg to all pregnant women and vaccination of their babies at birth has been recommended widely, yet it is not a routine practice in most health settings of Ethiopia [10].

It was estimated that over 5 million people are living with chronic HBV infection among the general population of Ethiopia [6]. However, data on the epidemiology of HBV infection in Ethiopia are limited, though few studies were conducted in the northern regions of the country. Using survey based questioners of Ethiopian blood donors, one study indicated that 8% and second 14.4% are infected by HBV in northern part of Ethiopia [12, 13]. Another study carried in Dessie [14] and Bahir Dar City [15] reported the prevalence of 4.9% and 3.8% among pregnant women, respectively. Other study from the same area also showed that 7.3% of sexually transmitted infections among women attending antenatal care were accounted for HBV infection [16]. These few studies indicated that hepatitis B infection among pregnant women raise a public health concern. The magnitude of the problem is not yet addressed in many parts of Ethiopia that include the Eastern Region. Different reports showed that the associated factors with HBV infections varied from region to region [14, 15, 16, 17]. Thus, determining the sero-prevalence and associated factors with HBV infection in different geographical setting is paramount to design appropriate preventive measures. Therefore, this study focused to determine the prevalence of HBV infection and associated factors among pregnant women attended antenatal care clinic in Deder Hospital, Eastern Ethiopia.

## Methods and Materials

### Study Setting and Period

The study was conducted at Deder Hospital located in Deder Town, East Hararghe Zone, Oromia Regional State, Eastern Ethiopia that is about 458 km from the capital city of Ethiopia, Addis Ababa. The hospital serves as a referral for more than one million people coming from four Districts. It has 95 beds for inpatient and outpatient health care services including ANC for pregnant women. In average, around 2100 pregnant women visit the ANC clinic per a year for routine pregnancy check-up services that include the assessment of pre-existing health conditions (screening for anemia, syphilis, HIV), vaccination, nutrition counseling, micronutrient supplementation and early detection of pregnancy related complications. The study was conducted between 18 March 2015 and 15 May 2015.

### Study Population and Sample Size

The study population was all consecutive pregnant women who attended ANC clinic for check-up services at Deder Hospital during the study period. Sample size was statistically calculated based on single population proportion formula by taking 7.3% prevalence of HBV infection from previous study [16], desired precision of 3%, a 95% confidence level and 10% non- response rate. Finally, the calculated sample size was 318.

A total of 338 pregnant mothers, who were eligible for the study, attended the ANC clinic during the study period. However, twenty of them were excluded because they refused to give blood sample and disagreed to participate in the study. Accordingly, the desired sample size, 318 pregnant women were included in the study.

### Sampling Procedure

As a routine service delivery procedure of the ANC clinic, pregnant women were triaged and assigned to the clinic. According to the sequence of their registration, they were allowed to enter into the ANC room for routine follow-up care one by one. Meanwhile, after the purpose of the study was explained to the subjects, they were asked to participate voluntarily in the study. All pregnant women with confirmed pregnancy were enrolled in this study. Pregnant mothers who attended the clinic for more than one visit during the study period were excluded. To avoid this, a unique mark was put on cards of all enrolled mothers. Accordingly, pregnant women who attended the ANC clinic were consecutively enrolled until the desired sample size was attained. Information on socio-demographic and other pertinent data was collected by trained data collectors using a pre-tested standard questionnaire adapted from the WHO “protocol for assessment of hepatitis B infection in antenatal patients” [18].

### Blood Sample Collection, Transportation and Processing

Three milliliters (ml) of venous blood was collected using plain tube by an experienced laboratory technologist from each study participant. Afterwards, the blood was allowed to clot at room temperature for about 30 minutes as recommended by testing kit’s manufacturer (Dialab GmbH, Austria). The tube was centrifuged at 800g for 20 minutes; serum was separated; transferred into cryo-tube and finally stored in a freezer at -20°C. The samples were transported to the Red Cross Society Blood Bank Laboratory, Harar Branch, Ethiopia in a cold box for serological test. Serological screening was done in the same day of arrival to control pre-analytical problem that could be occurred due to multiple freeze-thaw cycles of the serum. Screening for HBsAg was performed at Red Cross Society Blood Bank Laboratory using the Dialab^®^ HBsAg ELISA kit (Dialab GmbH, Austria) that can detect all HBV genotypes and subtypes. To maintain the quality of laboratory results, standard operating procedures (SOPs) of the test kit’s manufacturer was followed strictly. Positive and negative controls were run along with each batch of ELISA test kit.

### Method of Data Analysis

Data were entered into Epi-Data version 3.1 and transferred to SPSS version 16 for analysis. Descriptive statistical tests such as proportion and mean were used to compute the socio-demographic, behavioral and the outcome variable. Binary logistic regression analysis was used to determine the association between explanatory variables and the outcome variable with odds ratio at 95% CI. All explanatory variables with P-value ≤ 0.2 in the bivariate analysis were included in multivariate logistic regression model. P-value ≤ 0.05 in multivariate analysis was considered as statistical significance.

### Ethical Considerations

This study obtained Ethical clearance from Institutional Health Research Ethics Review Committee (IHRERC) of the College of Health and Medical Sciences of Haramaya University. All study participants were informed about the study and assured about the confidentiality, protection and anonymity of data. Written consent was obtained from each study participant prior to data collection and they participated voluntarily in the study. Laboratory results of HBsAg were reported to the health facility for further management of infected pregnant women. All HBsAg positive mothers were referred to physician and followed by testing their liver function in hospital’s laboratory. However, only supportive cares were offered because treatment guideline and specific drugs for HBV infections are not available in most health facilities of Ethiopia.

## Results

### Socio-demographic Characteristics of the Study Participants

A total of 318 pregnant women participated in this study with a response rate of 100%. The age of the study participants range between 15 and 40 years with a mean age of 25 years. More than half of the study participants, 181 (56.9%) were living in rural areas. Two hundred ninety nine (94%) of them were married and 274(86.2%) were Oromo by ethnicity. One hundred thirty two (41.5%) of the participants did not attend formal education. One hundred seventy (53%) were housewives and the others were teachers and merchants by occupation (Table 1).

**Table 1:** Socio-demographic characteristics of pregnant women attending ANC clinic at Deder hospital, Eastern Ethiopia, 2015 (N = 318).

Characteristic		Number	Percentage (%)
**Age (in year)**	15–19	88	27.6
	20–24	112	35.2
	25–29	66	20.8
	≥30	52	16.4
**Residence**	Urban	137	43.1
	Rural	181	56.9
**Marital Status**	Single	5	1.6
	Married	299	94
	Divorced	7	2.2
	Widowed	7	2.2
**Ethnicity**	Oromo	274	86.2
	Amhara	30	9.4
	Gurage	9	2.8
	Siltie	5	1.6
**Educational Status**	No formal education	132	41.5
	Primary school	119	37.4
	Secondary & above	67	21.1
**Occupation**	Farmer	88	28
	Health worker	8	3
	Housewife	170	53
	Daily Laborers	14	4
	Others	38	12

### Prevalence of Hepatitis B Virus Infection

In this study, the overall prevalence of HBsAg was 6.9% (22/318). Higher prevalence, 8% (9/112), was observed in the age group of 20–24 years. The prevalence of HBV infection increased with educational status of study participants throughout the entire sub-category. The highest prevalence of 37.5% was recorded in health care worker pregnant women. (Table 2).

**Table 2:** Prevalence of HBV among pregnant women attending ANC clinic at Deder Hospital, Eastern Ethiopia 2015 (N = 318).

Variable		HBsAg Status	
		Positive n (%)	Negative n (%)
**Age (in year)**	15–19	5(5.7)	83(94.3)
	20–24	9(8)	103(92)
	25–29	5(7.6)	61(92.4)
	≥30	3(5.8)	49(94.2)
**Residence**	Urban	12(8.8)	125(91.2)
	Rural	10(5.5)	171(94.5)
**Marital Status**	Single	1(20)	4(80)
	Married	20(6.7)	279(93.3)
	Divorced	0(0)	7(100)
	Widowed	1(14.3)	6(85.7)
**Ethnicity**	Oromo	19(6.9)	255(93.1)
	Amhara	3(10)	27(90)
	Gurage	0(0)	9(100)
	Siltie	0(0)	5(100)
**Educational Status**	No formal education	7(5.3)	125(94.7)
	Primary school	8(6.7)	111(93.3)
	Secondary & above	7(10.4)	60(89.6)
**Occupation**	Farmer	6(6.8)	82(93.2)
	Health worker	3(37.5)	5(62.5)
	Housewife	9(5.3)	161(94.7)
	Daily Laborers	1(7.1)	13(92.9)
	Others	3(7.9)	35(92.1)
**Gravidity**	Primigravida	3(4.1)	70(95.9)
	Multigravida	19(7.8)	226(92.2)
**Parity**	Nullipara	3(3.7)	79(96.3)
	Multipara	19(8.1)	217(91.9)
**History of blood transfusion**	Yes	0(0)	2(100)
	No	22(6.9)	294(93.1)
**HIV sero-status**	Positive	0(0)	0(0)
	Negative	22(6.9)	294(93.1)

### Factors Associated with Hepatitis B Virus Infection

Results of multivariate logistic regression analysis indicated that few predictor variables showed statistically significant association with HBV infections. Pregnant women who had a history of abortion were 10 times more likely of being infected by HBV than pregnant women who had no history of abortion. Having history of nose piercing were more likely to be infected (AOR 8.9; 95% CI 1.34–59.39) than their counterpart. Similarly, history of surgical procedure was significantly associated with HBV infection (AOR 13.3; 95% CI 1.7–103.8). Pregnant women who had history of multiple sexual partners were about 16 times more likely of being infected than those had no history of multiple sexual partners (Table 3).

**Table 3:** Statistical association of predictor variables with HBV infection, among pregnant women attending ANC clinic at Deder Hospital, Eastern Ethiopia, 2015, (N = 318).

Variables	HBsAg Status		Crude Odds Ratio (COR (95% CI))	Adjusted Odds Ratio	
	Positive n (%)	Negative n (%)		AOR (95% CI)	P-value
**Age (in year)**				*	
15–19	5(5.7)	83(94.3)	1		
20–24	9(8)	103(92)	1.450 (.468–4.494)		
25–29	5(7.6)	61(92.4)	1.361 (.377–4.908)		
>30	3(5.8)	49(94.2)	1.016 (.233–4.439)		
**Residence**				*	
Urban	12(8.8)	125(91.2)	1.642(0.688–3.920)		
Rural	10(5.5)	171(94.5)	1		
**Educational Status**				*	
No formal education	7(5.3)	125(94.7)	1		
Primary school	8(6.7)	111(93.3)	1.287(0.452–3.663)		
Secondary & above	7(10.4)	60(89.6)	2.083(0.699–6.208)		
**Occupation**				*	
Farmer	6(6.8)	82(93.2)	1		
Health worker	3(37.5)	5(62.5)	8.2(1.568–42.870)		
Housewife	9(5.3)	161(94.7)	0.764(0.263–2.220)		
Daily Laborers	1(7.1)	13(92.9)	1.051(0.117–9.453)		
Others	3(7.9)	35(92.1)	1.171(0.277–4.951)		
**Gravidity**				*	
Primigravida	3(4.1)	70(95.9)	1		
Multigravida	19(7.8)	226(92.2)	1.962(0.564–6.825)		
**Parity**				*	
Nullipara	4(4.8)	79(95.2)	1		
Multipara	18(7.7)	217(92.3)	1.638(0.538–4.989)		
**Home delivery by traditional birth attendant**				*	
Yes	13(8.2)	146(91.8)	1.484(0.616–3.578)		
No	9(5.7)	150(94.3)	1		
**History of abortion**					
Yes	17(22.7)	58(77.3)	13.952(4.943–39.378)	10.9(2.2–53.9)	0.003
No	5(2.1)	238(97.9)	1	1	
**Ear Piercing**				*	
Yes	21(7.6)	254(92.4)	1.654(0.373–7.335)		
No	1(2.3)	42(97.7)	1		
**Nose Piercing**					
Yes	14(63.6)	8(36.4)	30.00(10.042–89.625)	8.9(1.3–59.39)	0.025
No	8(2.7)	288(97.3)	1	1	
**Tattooing**					
Yes	11(26.2)	31(73.8)	8.548(3.424–21.339)	2.9(0.59–15.1)	0.185
No	11(4)	265(96)	1	1	
**Traditional Uvulectomy or/and tonsillectomy**					
Yes	17(13.1)	113(86.9)	5.506(1.977–15.336)	3.2(0.65–16.16)	0.152
No	5(2.7)	183(97.3)	1	1	
**History of hospital or HC admission**					
Yes	17(27)	46(73)	18.478(6.495–52.569)	3.2(0.73–14.45)	0.122
No	5(2)	250(98)	1	1	
**History of surgical Procedure**					
Yes	7(46.7)	8(53.3)	16.8(5.376–52.502)	13.3(1.7–103.8)	0.014
No	15(5)	288(95)	1	1	
**History of tooth extraction**					
Yes	6(14.6)	35(85.4)	2.796(1.026–7.62)	1.8(0.32–9.72)	0.514
No	16(5.8)	261(94.2)	1	1	
**History of sexually transmitted infection**					
Yes	14(36.8)	24(63.2)	19.833(7.566–51.992)	3.2(0.7–14.29)	0.129
No	8(2.9)	272(97.1)	1	1	
**History of multiple sexual partners**					
Yes	16(36.4)	28(63.6)	25.524(9.242–70.486)	16.8(3.2–87.9)	0.001
No	6(2.2)	268(97.8)	1	1	

1 = reference category,

* = P.Value greater than 0.2 in bivariate analysis,

HC = health center

## Discussion

In this study, we found that the prevalence of HBsAg among study participants was 6.9%. According to established criterion, the prevalence of HBsAg among pregnant women in this study area can be classified as “high inter-mediate” endemicity area [1]. This finding is similar with a prevalence of 6.1% reported in Southern Ethiopia [19], 6% in Addis Ababa [20] and 7.3% in Gondar, Northwest Ethiopia [16]. But, relatively it is higher than 4.9%, 4.4%, 4.3% and 3.8% of prevalence which were reported from Dessie [14], Felege Hiwot [21], Arba Minch [22] and Bahir Dar city [15], respectively. However, it is lower than earlier survey of Ethiopian blood donors (8%) [12] and 14.4% [13] of HBV sero-prevalence from northern part of Ethiopia. On the other hand, in comparison with other countries, higher results (16.6%) were reported in Nigeria [23] and (10.6%) in Ghana [24]. Whereas, lower prevalence, 1.5%, 1.6% and 4% were reported in Libya, Algeria and Tunisia, respectively [25]. This variation might be due to differences in sampling method, geographical variation, differences in cultural practices, sexual behavior and practices, and differences in the test methods employed to detect HBV infection.

In the present study, the prevalence of HBsAg was high among the age group of 20–24 years followed by 25–29. This finding is consistent with a report in Nigeria [26]. The possible explanation for this finding could be that women in these age groups are more sexually active and they may have higher chance of multiple sexual partners.

In fact our study demonstrated that history of abortion, nose piercing, surgical procedure and history of multiple sexual partners were significant predictors of HBV infection. Women with a history of abortion had a chance of 11 times (AOR10.9, 95%CI 2.2–53.9) to develop HBV infection compared to their counterpart. Similar results were reported from Jimma [27], Arba Minch [22], Addis Ababa [20] and Dessie, Ethiopia [14]. This high incidence of infection could be attributed to poor practices of infection prevention control during abortion and related activities. Moreover, women with a history of multiple sexual partners were 17 times (AOR 16.7, 95%CI3.2–87.9) more likely to develop HBV infection compared with those having single partner. Similar findings reported in Addis Ababa, Ethiopia [28]; and in Nigeria [29]. This finding may be explained as since hepatitis B is blood born virus; blood, semen and other body fluids are common source of infection that sexual contacts serve as a mode of transmission. Thus, sexually active women have a higher chance of getting the infection especially those who have the history of multiple sexual partners.

In this study, history of surgical procedure and nose piercing were statistically associated with HBV infection. Similar results were reported in Dessie [14] and Bahir Dar, Ethiopia [15]. But, other reports from Nigeria [29]; Mauritania [30] and Yemen [31] were contradicted with our findings. The difference could be attributed to variations in sample size, study period and poor practices of infection prevention strategies. Interestingly we found that factors such as educational status, tattooing, home delivery, blood transfusion and history of tooth extraction were not statistically associated with HBV infection that are consistent with the findings reported in Nigeria [26] and in Sudan [32] but inconsistent with other studies [14, 15, 33]. These differences could be due to variations in sample size, duration of study and safety precautions being taken.

Ethiopia is among the countries with high burden of HIV infection and it is located in a region classified as high endemic area for HBV [34]. Due to that HIV and HBV share the same modes of transmission; the likelihood of HBV/HIV co-infection is anticipated. But, in this study, positive HIV test result was not observed in all study participants. One of the possible reasons could be the drawback of secondary data source because HIV test results of women were collected from the antenatal care clinic’s registration book. However, previous studies reported the co-infection rate of 22.6% [14]; 19% [15] from Northern and 0.6% [19] from Southern Ethiopia.

In this study very important predictor variables are significantly associated with the HBV infection despite high odds ratio and wide confidence intervals were observed statistically. These might be due to the small sample size and few numbers of cases (positive for HBsAg) in our study. Other possible causes for these huge statistical results such as confounding effects, multicollinearity and the validity of regression model were addressed at the stage of study design and during data analysis. Likewise, similar numerical problems were reported in other comparable studies [14, 20]. For the purpose of this study, only HBsAg was determined which tells about active infection rather than total prevalence. But screening for other serological markers such as anti-HBs and anti-HBc antibodies would have great potential to determine the overall prevalence of HBV infection. We neither tested for anti-HBc (IgM), nor followed up women to determine if they had a chronic or an acute infection. Moreover, we could not determine the extent of perinatal transmission of HBV; because the prevalence of HBeAg or the viral load was not assessed among HBsAg sero-positive pregnant mothers due to lack of reagent kits and resource constraints. These all should be considered as the limitations of this study.

## Conclusions and Recommendations

The overall prevalence of HBV infection among pregnant women in Deder, Eastern Ethiopia is high-intermediate (6.9%) endemic area according to the WHO classification criteria. This finding suggests that the vertical transmission of HBV infection may be a serious public health problem in Deder district. Factors such as history of abortion, nose piercing, surgical procedure and history of multiple sexual partners were significantly associated with HBV infection. Thus, to decrease the prevalence of this viral infection, we recommend that health education programs on the mode of HBV transmission, high-risk behaviors and methods of preventions should be instituted at antenatal care clinics to raise the awareness of mothers. It is also advisable to implement nosocomial infection prevention strategies to prevent the transmissions of HBV through health care related activities such as surgical procedures. Furthermore, all pregnant women should be screened for HBV, treated if necessary to reduce their viral loads and their children vaccinated at birth with the single-dose hepatitis B vaccine to break the cycle of mother-to-child transmission.

## References

[1] World Health Organization. Guidelines for the prevention, care and treatment of persons with chronic hepatitis B infection. Geneva; 3, 2015, WC- 536. 26225396

[2] Adibi P, Akbari L, Kahangi LS, Abdi F. Health-State Utilities in Liver Cirrhosis: A Cross- sectional Study. Int J Prev Med. 2012; 3: 94–101. PMC3399292 22826776

[3] Verma R, Khanna P, Prinja S, Rajput M, Chawla S, Bairwa M. Hepatitis B Vaccine in national immunization schedule: a preventive step in India. Hum Vaccin. 2011, 7: 1387–1388. 10.4161/hv.7.12.17878 22134433

[4] Pungpapong S, Kim WR, Poterucha JJ. Natural history of HBV infection: an update for clinicians. Mayo Clin Proced. 2007, 82:967–975. 10.4065/82.8.967 17673066

[5] World Health Organization. Hepatitis B Fact sheet no 204. Geneva; 2015 http://www.who.int/mediacentre/factsheets/fs204/en/

[6] Schweitzer A, Horn J, Mikolajczyk RT, Krause G, Ott JJ. Estimations of worldwide prevalence of chronic hepatitis B virus infection. A systematic review of data published between 1965 and 2013. Lancet; Published online July 28, 2015 10.1016/S0140-6736(15)61412-X. 26231459

[7] Global Burden of Disease (GBD) Mortality and Causes of Death Collaborators. Global, regional and national age-sex specific all-cause and cause-specific mortality for 240 causes of death, 1990–2013. A systematic analysis for the Global Burden of Disease Study. Lancet. 2015, 385:117–71. 10.1016/S0140-6736(14)61682-2 25530442 PMC4340604

[8] World Health Organization. Hepatitis B vaccines Weakly Epidemiolo Rec. 2009, 84: 405–419.

[9] Goldstein ST, Zhou F, Hadler SC, Beth P Bell BP, Eric E Mast EE, Margolis HS. A mathematical model to estimate global hepatitis B disease burden and vaccination impact. Int J Epidemiol. 2005, 34(6):1329–1339. 16249217 10.1093/ije/dyi206

[10] Federal Democratic Republic of Ethiopia. National expanded programme on immunization comprehensive multi-year plan 2011–2015. Addis Ababa: Fedral Ministry of Health, 2010.

[11] Stewart RD, Sheffield JS. Hepatitis B Vaccination in Pregnancy in the United States. Department of Obstetrics & Gynecology, University of Texas Southwestern Medical Center 2013, 167–173;

[12] Kefene H, Rapicetta M, Rossi GB. Ethiopian National HBV Study. J Med Virol. 1988, 24:75–84. 3339335 10.1002/jmv.1890240110

[13] Gebreselassie L. Occurrence of HBsAg and its antibody in Ethiopian blood donors. Ethiopia Medical Journal. 1983, 21: 205–208. 6628366

[14] Baye G, Mohammed S, Abate A. Sero-prevalence of HBV and HCV Infections Among Pregnant Women Attending Antenatal Care Clinic at Dessie Referral Hospital, Ethiopia. Advances in Life Sciences and Health. 2014, 1(2): 109–120.

[15] Zenebe Z, Mulu W, Yimer M, Abera B. Sero-prevalence and risk factors of hepatitis B virus and human immunodeficiency virus infection among pregnant women in Bahir Dar city, Northwest Ethiopia. BMC Infec Dis. 2014,14:118. 24580859 10.1186/1471-2334-14-118 PMC3942511

[16] Tiruneh M. Seroprevalence of multiple sexually transmitted infections among antenatal clinic attendees in Gondar Health Center, northwest Ethiopia. Ethiop Med J. 2008, 46(4):359–66. 19271400

[17] Tegegne D, Desta K, Tegbaru B, Tilahun T. Seroprevalence and transmission of Hepatitis B virus among delivering women and their new born in selected health facilities, Addis Ababa, Ethiopia: a cross-sectional study. BMC Research Notes. 2014,7:239 10.1186/1756-0500-7-239 24731794 PMC3991867

[18] World Health Organization. Expanded Programme on Immunization Protocol for Assessing Prevalence of Hepatitis B Infection in Antenatal Patients. Geneva 1990,WHO/EPI/GEN/90.6.

[19] Ramos JM, Toro C, Reyes F, Amor A, Gutiérrez F. Seroprevalence of HIV-1, HBV, HTLV-1 and Treponema pallidum among pregnant women in a rural hospital in Southern Ethiopia. J Clin Virol. 2011, 51(1):83–85. 10.1016/j.jcv.2011.01.010 21330196

[20] Desalegn Z, Wassie L, Beyene HB, Mihret A, Ebstie YA. Hepatitis B and human immunodeficiency virus co-infection among pregnant women in resource-limited high endemic setting, Addis Ababa, Ethiopia: implications for prevention and control measures. Eur J Med Res. 2016 4 14;21:16 10.1186/s40001-016-0211-3 27075475 PMC4831185

[21] Molla S, Munshea A, Nibret E. Seroprevalence of hepatitis B surface antigen and anti HCV antibody and its associated risk factors among pregnant women attending maternity ward of Felege Hiwot Referral Hospital, northwest Ethiopia: a cross-sectional study. Virol J. 2015 12 2;12:204 10.1186/s12985-015-0437-7 26626263 PMC4667425

[22] Yohanes T, Zerdo Z, Chufamo N. Seroprevalence and Predictors of Hepatitis B Virus Infection among Pregnant Women Attending Routine Antenatal Care in Arba Minch Hospital, South Ethiopia. Hepat Res Treat. 2016;2016:9290163. 10.1155/2016/9290163 PMC4745621 26904281

[23] Kolawole OM, Wahab AA, Adekanle DA, Sibanda T, Okoh A. Seroprevalence of hepatitis B surface antigenemia and its effects on hematological parameters in pregnant women in Osogbo, Nigeria. Virology J. 2012, 9:317. 23268985 10.1186/1743-422X-9-317 PMC3546843

[24] Cho Y, Bonsu G, Ampaw AA, Mills GN, Nimo JA, Park JK, et al The Prevalence and Risk Factors for Hepatitis B Surface Ag Positivity in Pregnant Women in Eastern Region of Ghana. Gut and Liver J. 2012, 6(2):235–240. 10.5009/gnl.2012.6.2.235 PMC3343163 22570754

[25] Gasim GI, Murad IA, Adam I. Regional Review of Hepatitis B and C virus infections among pregnant women in Arab and African countries. J Infect Dev Ctries. 2013, 7(8):566–578; 10.3855/jidc.3243 23949291

[26] Eke AC, Eke UA, Okafor CI, Ezebialu IU, Ogbuagu C. Prevalence, correlates and pattern of hepatitis B surface antigen in a low resource setting.Virol Journal. 2011, 8(12):01–08 10.1186/1743-422X-8-12 PMC3027130 21226907

[27] Awole M, Gebre-Selassie S. Seroprevalence of HBsAg and its risk factors among pregnant women in Jimma, Southwest Ethiopia. Ethiop J Health Dev.2005, 19(1):45–50.

[28] Duncan ME, Tibaus G, Pelger A. Prevalence and significance of sexually transmitted diseases among women attending clinics in Addis Ababa. Ethiop Journal Health Development. 1995, 9:31–40.

[29] Rabiu KA, Akinola OL, Adewunmi AA, Omolilu OM, Ojo TO. Risk factors for hepatitis B virus infection among pregnant women in Lagos, Nigeria. Acta Obstetricia Gynecological. 2010, 89: 1024–1028. 10.3109/00016349.2010.482580 20636241

[30] Mansour W, Malick FZ, Sidiya A, Ishagh E. Prevalence, risk factors and molecular epidemiology of hepatitis B and hepatitis delta virus in pregnant women and in patients in Mauritania. J Med Virol. 2012, 84: 1186–1198. 10.1002/jmv.23336 22711346

[31] Murad EA, Babiker SM, Gasim GI, Rayis DA, Adam I. Epidemiology of hepatitis B and hepatitis C virus infections in pregnant women in Sana’a, Yemen. BMC Pregnancy and Child birth. 2013,13:127. 10.1186/1471-2393-13-127 PMC3684507 23758990

[32] Elsheikh RM, Daak AA, Elsheikh MA, Karsany MS, Adam I. Hepatitis B virus and hepatitis C virus in pregnant Sudanese women. Virol J. 2007,4:104–106 10.1186/1743-422X-4-104 17958904 PMC2116999

[33] Yakasai IA, Ayyuba R, Abubakar IS, Ibrahim SA. Sero-prevalence of hepatitis B virus infection and its risk factors among pregnant women attending antenatal clinic at Aminu Kano teaching hospital, Kano, Nigeria. J Basic Clin Reprod Sci. 2012, 1:49–55.

[34] Negero A, Sisay Z, Medhin G. Prevalence of Hepatitis B surface antigen (HBsAg) among visitors of Shashemene General Hospital voluntary counseling and testing center. BMC Research Notes. 2011, 4(6):3–5. 21303563 10.1186/1756-0500-4-35 PMC3045955

